# Author Correction: Eya3 partners with PP2A to induce c-Myc stabilization and tumor progression

**DOI:** 10.1038/s41467-018-06265-3

**Published:** 2018-09-17

**Authors:** Lingdi Zhang, Hengbo Zhou, Xueni Li, Rebecca L. Vartuli, Michael Rowse, Yongna Xing, Pratyaydipta Rudra, Debashis Ghosh, Rui Zhao, Heide L. Ford

**Affiliations:** 1 Department of Biochemistry and Molecular Genetics, University of Colorado Denver Anschutz Medical Campus, Aurora 80045, CO USA; 2 Department of Pharmacology, University of Colorado Anschutz Medical Campus, Aurora 80045, CO USA; 3 Cancer Biology Program, University of Colorado Anschutz Medical Campus, Aurora 80045, CO USA; 4 Molecular Biology Program, University of Colorado Anschutz Medical Campus, Aurora 80045, CO USA; 5 McArdle Laboratory for Cancer Research, School of Medicine and Public Health, University of Wisconsin-Madison, Madison 53705, WI USA; 6 Department of Biostatistics and Informatics, University of Colorado Anschutz Medical Campus, Aurora 80045, CO USA

Correction to: *Nature Communications*, 10.1038/s41467-018-03327-4, published online 13 March 2018

In the original version of this Article, the title of the legend to Fig. 7 incorrectly read ‘Knockdown of B55α increases breast cancer metastasis’ instead of ‘Knockdown of B55α decreases breast cancer metastasis’. This has now been corrected in both the PDF and HTML versions of the Article.

