# Supplementary material for: Eya3 partners with PP2A to induce c-Myc stabilization and tumor progression
Source: Nat Commun. 2018 Mar 13;9:1047. doi: 10.1038/s41467-018-03327-4 (PMC5849647; doi:10.1038/s41467-018-03327-4)
Supplement: Supplementary file 1 — Supplementary Information [file 41467_2018_3327_MOESM1_ESM.pdf]

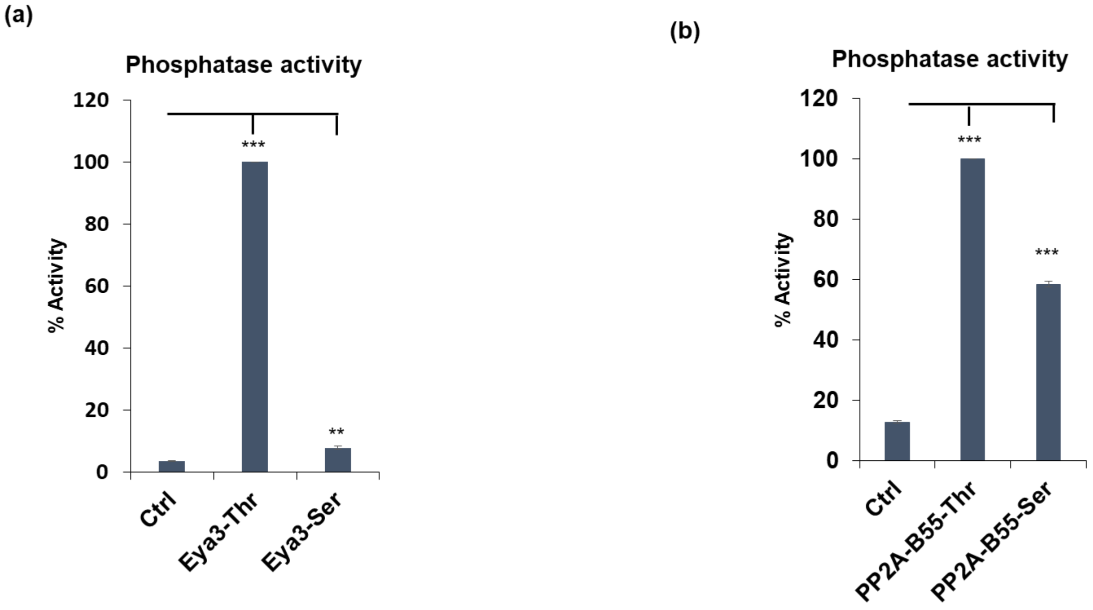

Supplementary Figure 1. Thr and Ser phosphatase activity of Eya3 and PP2A-B55α. (a). Eya3 was purified from HEK293FT cells and Thr and Ser phosphatase activities were assessed through a malachite green assay using a phospho-Thr peptide (KRpTIRR) or phospho-Ser peptide (RRApSVA) as substrate, respectively. The results show Eya3 purified from HEK293FT cells has much higher Thr than Ser phosphatase activity. (b) The PP2A-B55α complex was assembled using equal amount of purified GST-Aα, his-B55α and his-Cα prior to evaluating its Thr and Ser Phosphatase activity. Our results show that PP2A-B55α has both Thr and Ser Phosphatase activity, although its Thr phosphatase activity is stronger. Error bars: standard deviation, n=3. The p-value is calculated using one-way ANOVA followed by Tukey Test, **$p<0.01$, and ***$p<0.001$.

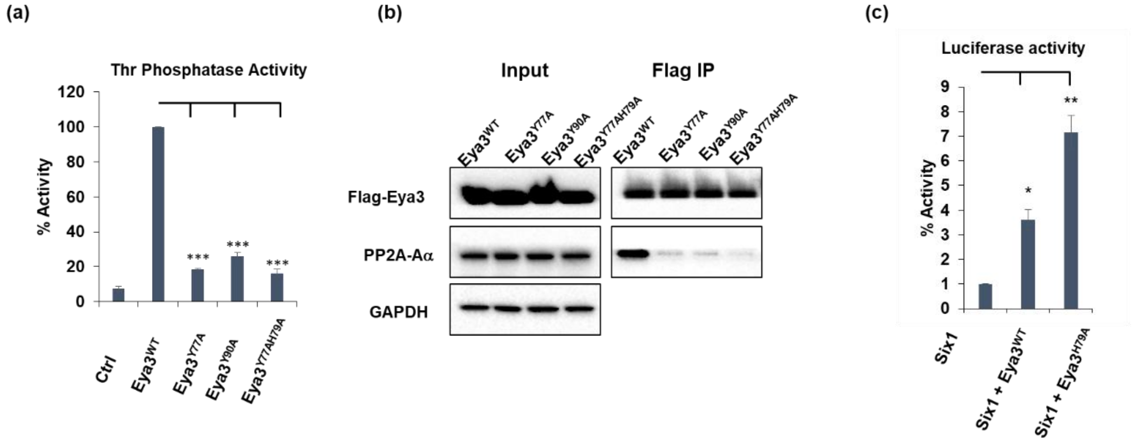

Supplementary Figure 2. **Characterization of Eya3 mutants.** (a) Eya mutants (Eya3^{Y77A}, Eya3^{Y90A} and Eya3^{Y77AH79A}) purified from HEK293FT cells have lower Thr Phosphatase activity compared to WT Eya3 (Eya3^{WT}), as shown using a malachite green assay and a phospho-Thr peptide (KRpTIRR) as substrate. Eya3 WT activity is set to 100%.The p-value is calculated using one-way ANOVA followed by Tukey Test, ***p<0.001. (b) Eya mutations previously found to be Thr phosphatase dead (Y77A, Y90A and Y77AH79A) show a decreased association with PP2A compared to WT, as seen using Flag-IP followed by Western blot analysis. (c) MEF3 luciferase reporter analysis shows the transcription activating potential of the Six1 protein alone and Six1/Eya3 protein complexes: Six1, Six1+ Eya3^{WT} and Six1+ Eya3^{H79A}. The H79A mutant of Eya3 does not diminish transcription mediated by the Six1/Eya3 complex. Error bars: standard deviation, n=3. The p-value is calculated using one-way ANOVA followed by Tukey Test, *p<0.05 and **p<0.01.

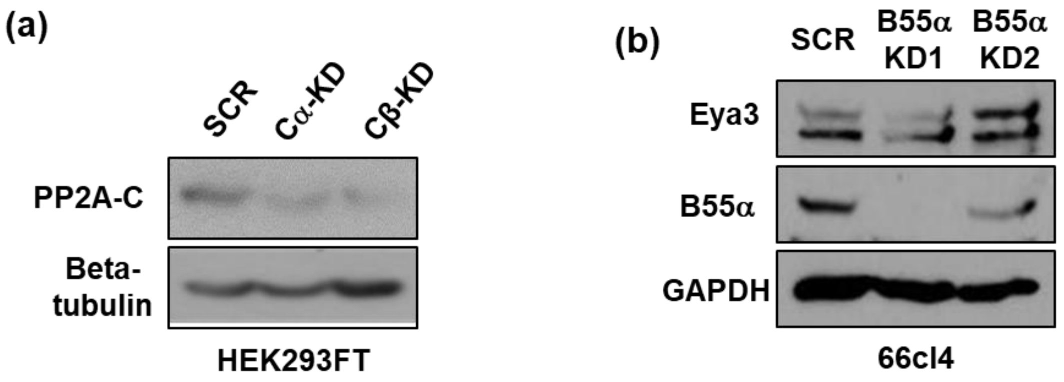

Supplementary Figure 3. Characterization of PP2A-C and B55α knockdowns in cell lines. (a) Western blot analysis shows the PP2A-C level in HEK293FT cells transduced with PP2A-Cα and Cβ shRNAs. (b) Endogenous PP2A-B55α in 66cl4 cells was knocked down using two different shRNAs, and scramble vector was used as a control. Western blot analysis shows Eya3 and PP2A-B55α levels in SCR or PP2A-B55α knockdown 66cl4 cells.

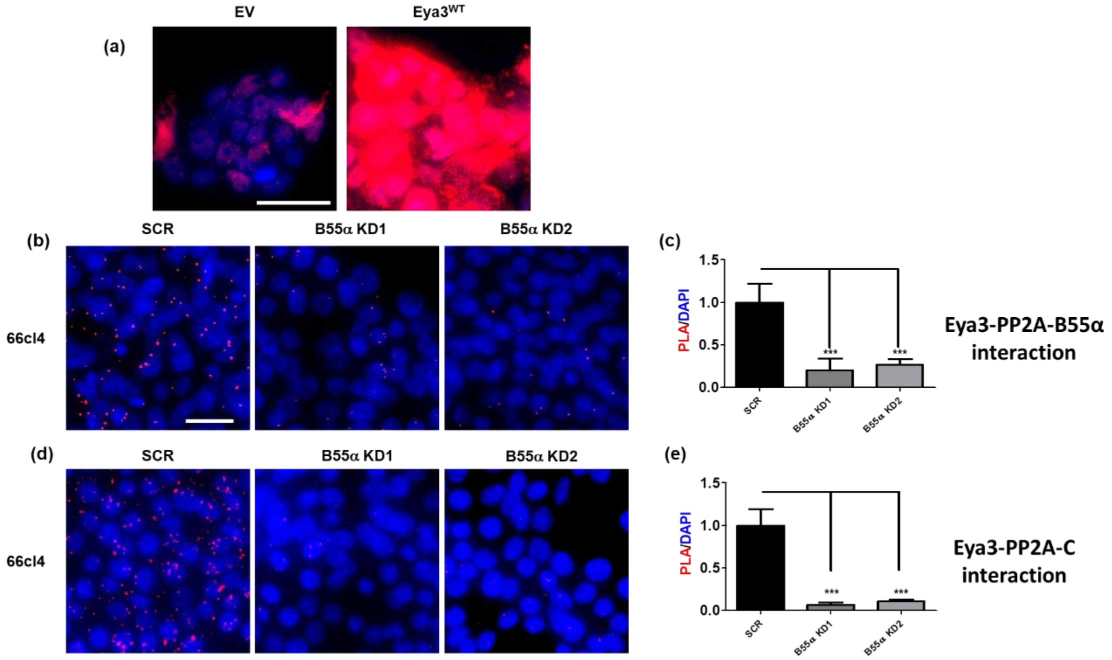

Supplementary Figure 4. Additional PLA images in various cell lines. (a) Representative images of PLA demonstrating the association between Eya3 and PP2A-C in HEK293FT transfected with EV or WT Eya3, scale bar: 20 μm. (b)-(c) & (d)-(e) Representative images of PLA examining the Eya3-PP2A-B55α and Eya3-PP2A-C interaction, respectively, in SCR or PP2A-B55α KD 66cl4 cells, scale bar: 20 μm. Graphs are quantification of the PLA/DAPI signal ratio shown in images, SCR is set to 1. P-value is calculated by one-way ANOVA followed by Tukey test, ***p<0.001, error bars: standard deviation, n=5.

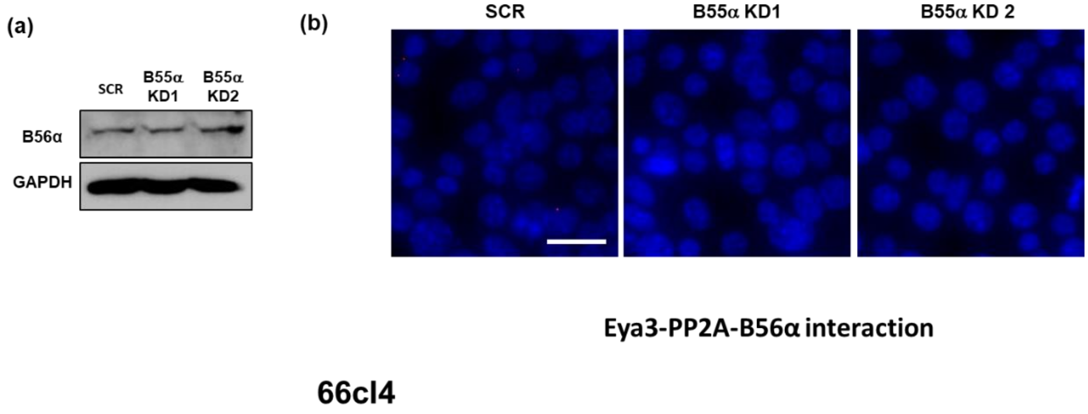

Supplementary Figure 5. B55α KD does not affect PP2A-B56α level, and Eya3 does not interact with PP2A-B56α. (a) Western blot analysis demonstrates the protein level of PP2A-B56α in SCR or KD 66cl4 cells. (b) Representative images of PLA examining the Eya3-PP2A-B56α interaction in SCR or PP2A-B55α KD 66cl4 cells, scale bar: 20 μm.

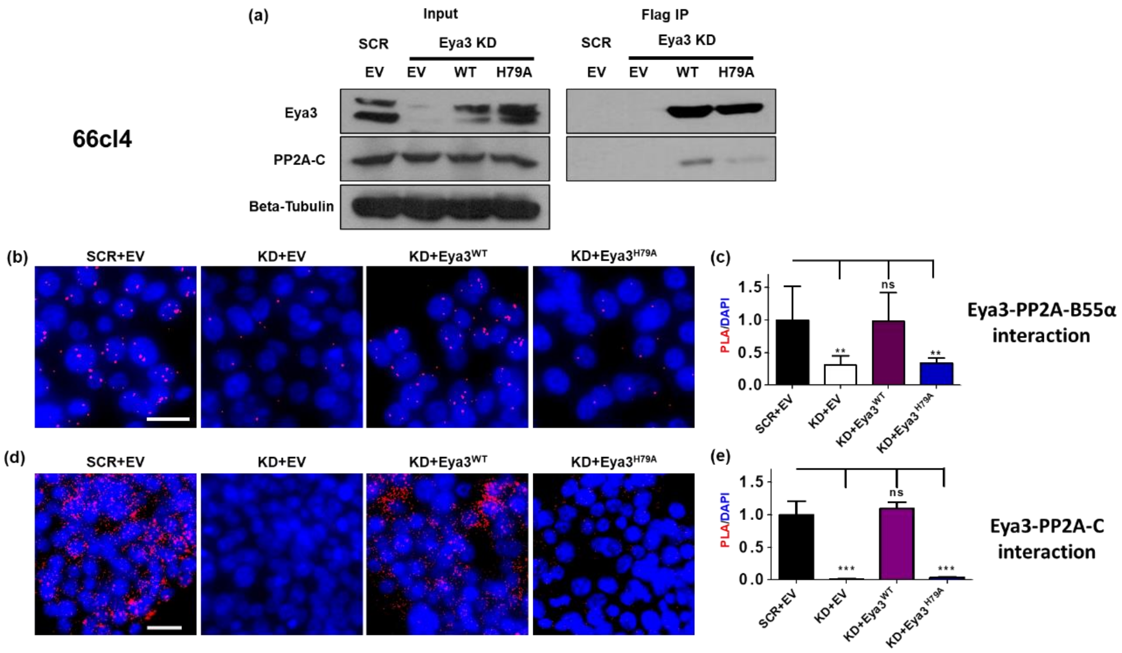

Supplementary Figure 6. Generation of Eya3 WT and H79A mutant addback 66cl4 cell lines. (a) Endogenous Eya3 in 66cl4 cells was knocked down using shRNA (SCR was used as control), and empty vector (EV), Flag-Eya3^{WT} or Flag-Eya3^{H79A} (Eya constructs carrying wobble mutations so as to not be knocked down by shRNA) were introduced. (a) Western blot analysis shows Eya3 levels in 66cl4 Eya3 addback cells (left panel). IP against Flag demonstrates the Eya3-PP2A-C interaction in 66cl4 Eya3^{WT}, which is dramatically reduced in Eya3^{H79A} addback cells (right panel). (b)-(c) & (d)-(e) Representative images of PLA examining the Eya3-PP2A-B55α and Eya3-PP2A-C interaction, respectively, in 66cl4 addback cells. Scale bar: 20 μm. Graphs are quantification of the PLA/DAPI signal ratio in images, with SCR+EV is set to 1. P-value is calculated using one-way ANOVA followed by Tukey test, ** $p<0.01$, ***$p<0.001$, error bars: standard deviations, n=5.

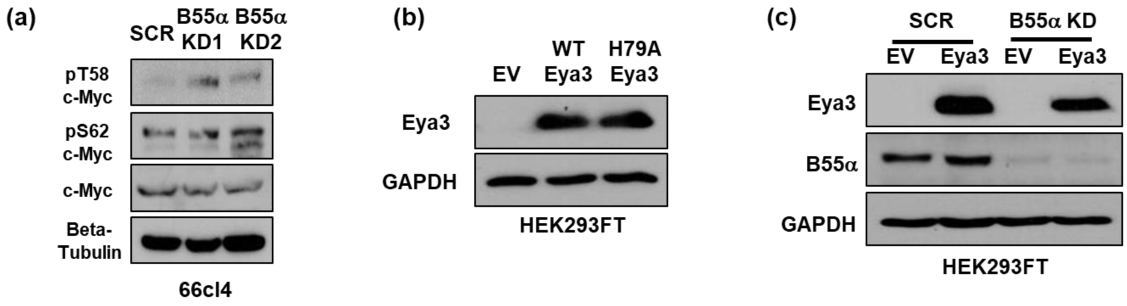

Supplementary Figure 7. Levels of key proteins in various KD or addback cell lines. (a) Western blot analysis shows pT58, pS62 and total c-Myc levels in SCR or PP2A-B55α knockdown 66cl4 cells. (b) Western blot analysis of Eya3 levels in HEK293FT cells transfected with EV, Eya3^{WT} or Eya3^{H79A} prior to performing the cycloheximide chase assay. (c) Western blot analysis of Eya3 and B55α in SCR or B55α KD HEK293FT cells transfected with EV or Eya3^{WT} prior to cycloheximide chase assay.

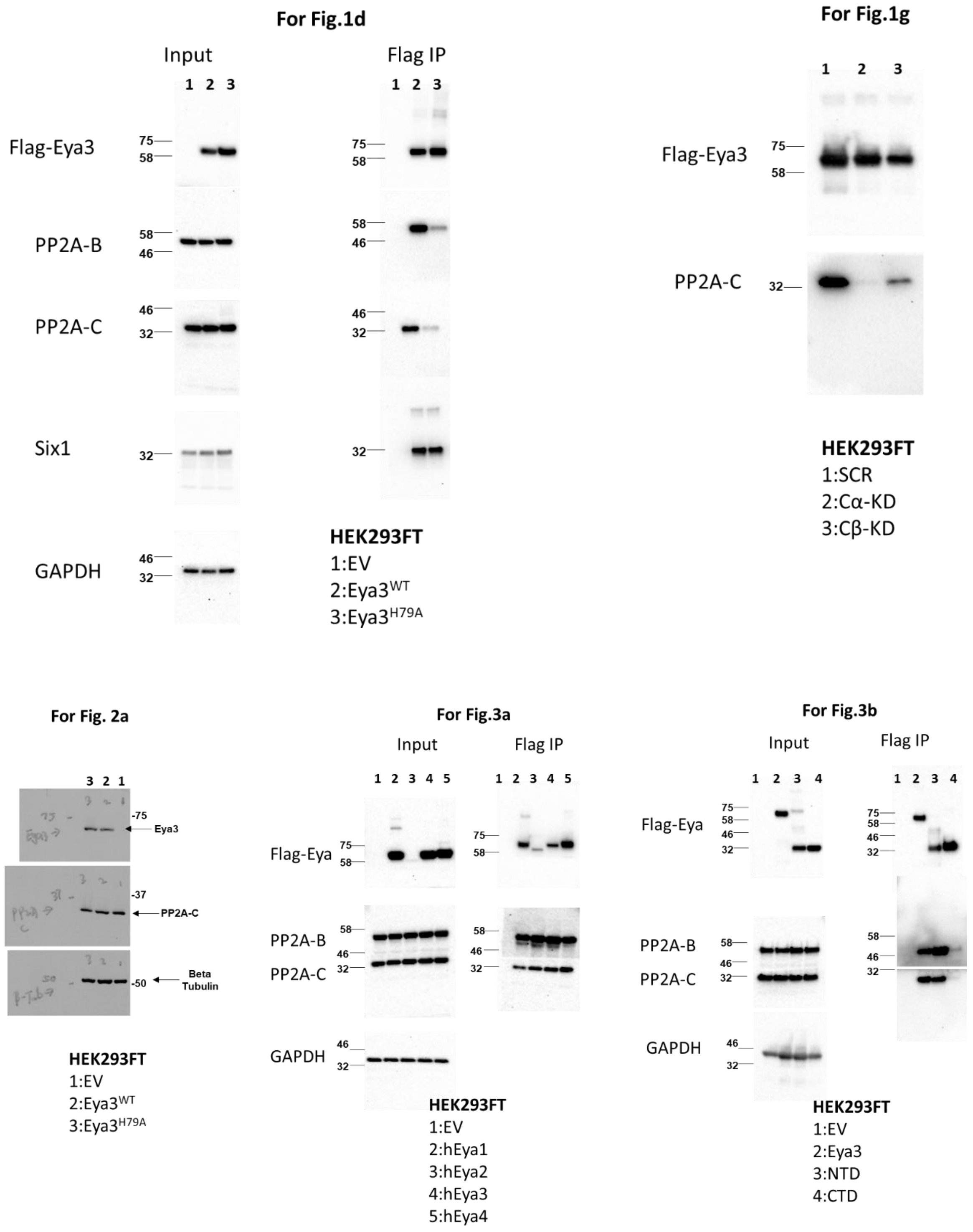

Supplementary Figure 8. Uncropped Western blots for various figures.

For Fig.4a

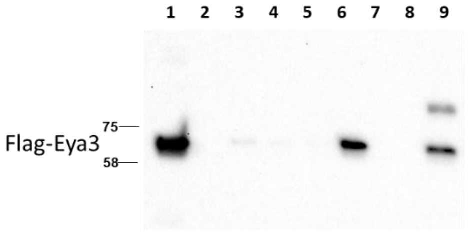

1: input
2: MW marker
3: Glutathione resin
4: GST-Aα
5: GST-Aα + Cα
6: GST-Aα + B55α + Cα
7: MW marker
8: Ni-NTA
9: His-B55α

For Fig.4b

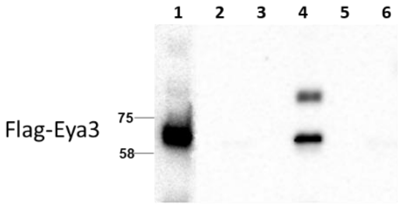

1: input
2: MW marker
3: Ni-NTA
4: His-B55α
5. Glutathione resin
6: GST-A56γ1

For Fig. 4c

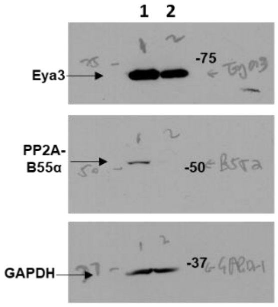

HEK293FT
1:SCR
2:B55α KD

For Fig. 4f

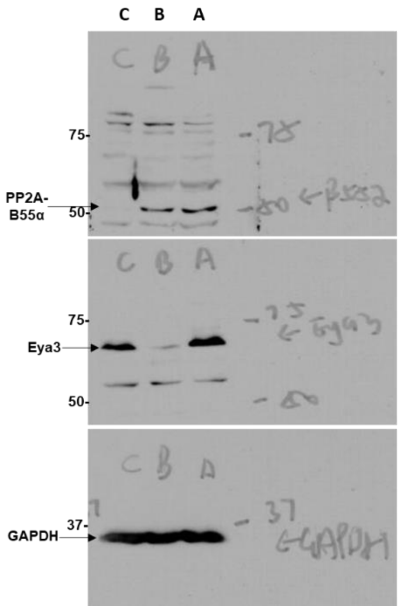

66cl4
A:SCR
B:Eya3 KD
C:B55α KD1

Supplementary Figure 8. Uncropped Western blots for various figures.

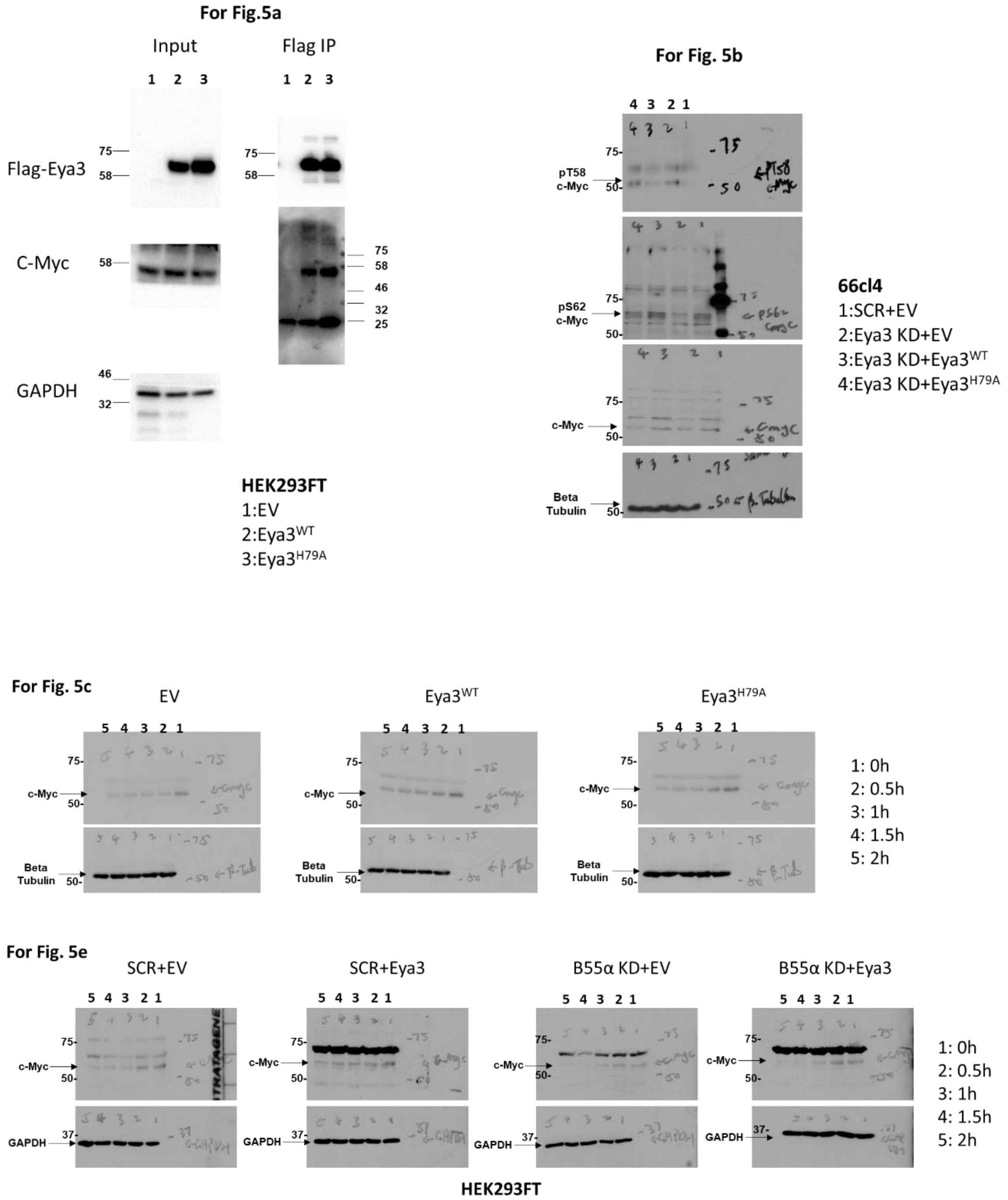

Supplementary Figure 8. Uncropped Western blots for various figures.
