# Supplementary material for: Eya3 partners with PP2A to induce c-Myc stabilization and tumor progression
Source: Nat Commun. 2018 Mar 13;9:1047. doi: 10.1038/s41467-018-03327-4 (PMC5849647; doi:10.1038/s41467-018-03327-4)
Supplement: Supplementary file 3 — Description of Additional Supplementary Files [file 41467_2018_3327_MOESM3_ESM.pdf]

Description of Additional Supplementary Files

File Name: Supplementary Data 1
Description: All proteins identified in mass spectrometry analyses of mouse Eya3 purified from HEK293FT cells.
